# Macro–Meso Damage Mechanism of Sandstone Under Wet–Dry Cycles: A Study Based on Nuclear Magnetic Resonance Technology

**DOI:** 10.3390/ma19061215

**Published:** 2026-03-19

**Authors:** Yuancheng Wei, Fujun Niu, Shu Zhu, Jin Zhang

**Affiliations:** 1 School of Civil Engineering and Transportation, South China University of Technology, Guangzhou 510641, China; 2 Yangtze River Delta Urban Wetland Ecosystem National Field Scientific Observation and Research Station, School of Environment and Geographic Sciences, Shanghai Normal University, Shanghai 200234, China; 3 Key Laboratory of Ministry of Education for Geomechanics and Embankment Engineering, Hohai University, Nanjing 210024, China

**Keywords:** wet–dry cycle, nuclear magnetic resonance, damage mechanism, mechanical properties, sandstone

## Abstract

Water level fluctuations in reservoir areas subject bank slopes to intense wet–dry cycles (WDCs), compromising rock mass stability. This study investigates the macro–meso damage evolution of yellow sandstone from the Wudongde Reservoir. Specimens subjected to 0–20 WDCs were analyzed using nuclear magnetic resonance (NMR) alongside Brazilian splitting, uniaxial, and triaxial compression tests. Results indicate that porosity increases linearly with WDC, rising from 6.12% to 17.61% after 20 cycles, driven by the transformation of micropores into macropores. Macroscopic mechanical parameters, particularly tensile strength and cohesion, exhibit significant exponential and sharp decay, respectively, while the internal friction angle remains relatively stable. Notably, increasing confining pressure effectively mitigates WDC-induced deterioration by inhibiting microcrack propagation. The damage mechanism is primarily attributed to the dissolution of clay binder and uneven mineral swelling/shrinkage, whereas the rigid mineral skeleton remains largely intact. These findings provide a theoretical basis for quantifying rock damage and predicting slope stability in complex hydrological environments.

## 1. Introduction

With the intensification of global warming, extreme climates trigger heavy rainfall and reservoir impoundment-discharge operations [1], leading to frequent and abrupt water level fluctuations in the reservoir area. Consequently, the reservoir bank slopes undergo intense wet–dry cycles (WDCs) [2,3]. During this process, the rock mass is continuously subjected to the alternating effects of immersion, scouring, undercutting, and drying, which not only induce slope deformation and instability but also significantly deteriorate its mechanical properties [4,5,6]. Accurately analyzing the deformation stability of rock slopes lies in clarifying the deterioration law of rock mass mechanical properties under WDC and further revealing the cumulative damage mechanism in this process [7,8,9]. Macroscopic analysis reveals the overall mechanical response and stability changes [10], while the mesoscopic perspective allows for a deep analysis of the intrinsic evolution of pore-fracture structures. The combination of both is the key to comprehensively explaining the damage effect induced by WDC. Therefore, conducting macro–meso research is crucial for understanding slope stability in reservoir drawdown zones and provides reliable theoretical support for disaster prevention.

To date, scholars have carried out a series of studies on rock mechanics and damage mechanisms of rocks under WDCs. Gao et al. conducted 40 WDC tests on schist and found that the shape of the stress–strain curve was independent of the number of cycles, while the uniaxial compressive strength of the rock decreased with the increase in cycle number [11]. Nan et al. confirmed through uniaxial and triaxial compression tests of sandstone that WDC and unloading effects weaken the mechanical properties of jointed sandstone, and the increase in confining pressure can alleviate the reduction amplitude of peak stress during unloading [12]. Zhang et al. found in shear creep tests of granite under WDCs that the number of cycles aggravates creep deformation and significantly increases the creep failure stress, duration and long-term strength [13]. Xu et al. studied the strength behavior of loess under WDCs and revealed the loess deterioration mechanism via SEM [14]. Cao et al. investigated the influence of WDCs with different salt solution concentrations on the pore-fracture structure of sandstone, and found that high-concentration salt solutions lead to an increase in rock pore size [15]. Xu et al. pointed out that the decay effect of peak strength of diorite is most significant under 1–3 WDCs, and the subsequent decay degree gradually slows down [16]. Dong et al. carried out 0–15 WDCs and conventional triaxial compression tests under a single confining pressure on Wuyishan red sandstone. Combined with SEM and X-ray diffraction, they found that long-term WDCs cause the dissolution of internal skeleton fillings in rocks, thereby reducing strength indexes [17]. Liu et al. compared and analyzed the uniaxial compressive results and multi-stage creep results of red sandstone without WDCs, under saturated state and under different WDC numbers, and found that the rock strength decreases most significantly under the first cycle [18]. In summary, existing studies have covered various rock types and discussed the influences of WDCs on rock mechanical properties and pore-fracture structures. Some studies have incorporated triaxial compression tests or micro-characterization techniques. However, there is still a lack of research on the pore structure evolution law of yellow sandstone under WDCs. Meanwhile, most existing WDC mechanical tests adopt uniaxial compression or conventional triaxial compression under a single confining pressure, failing to systematically explore the mechanical response of yellow sandstone under the coupling effect of confining pressure and WDC number.

As a non-destructive testing technique, nuclear magnetic resonance (NMR) boasts advantages of non-destructiveness, rapidity, and high precision [19], and has been widely applied in fields such as petroleum development and biomedicine [20,21,22]. Based on hydrogen-containing fluid detection, NMR can obtain quantitative parameters related to pore structure of rocks, including porosity and pore size distribution. In recent years, NMR has been relatively extensively used in the geotechnical engineering field. For instance, Long et al. studied the influence of injection rate on the mesoscopic structure of sandstone using a self-developed NMR and hydraulic fracturing test system [23]. Li et al. measured the pore state of granite after high-temperature water cooling using NMR, addressing the stability maintenance of high-temperature geothermal wellbores [24]. Li et al. analyzed the porosity of cement cylinders with different salt contents under different dry–wet cycle times via NMR [25]. Deng et al. examined the pore distribution of red sandstone from Jiangxi, China, under different initial saturations and wet–dry cycle times [26]. Taking the water storage and energy storage reservoir of abandoned mines as the research background, Yuan et al. analyzed the pore and composition changes of sandstone under acid WDCs by means of NMR, EDS, XRD and other techniques [27]. Obviously, researchers have characterized the changes in rock pore structures under different working conditions using NMR technology. However, studies that systematically correlate quantified pore data with the evolution parameters of rock mechanical properties under WDCs are relatively insufficient, making it difficult to reveal the deterioration mechanism of rock mechanical properties under the coupling effect of engineering stress and WDCs.

Based on existing research, typical slope sandstone from China’s Wudongde Reservoir was selected as the research object. Indoor WDC tests of sandstone were designed, and NMR core microscopic non-destructive testing was used to study the sandstone after WDCs, aiming to analyze the influence mechanism of WDCs on NMR T2 curves, porosity, and pore size distribution. Indoor Brazilian splitting tests, uniaxial compression tests, and conventional triaxial compression tests were carried out on the sandstone after WDCs to obtain the strength and deformation characteristics of sandstone under WDCs. Combined with the rock mechanics test results, pore variation data from NMR, and SEM image information, the macro–mesoscopic mechanical damage mechanism of yellow sandstone induced by WDCs is jointly revealed, which provides a basis for developing damage constitutive models that quantitatively characterize the damage evolution of rock materials in complex environments.

## 2. Test Methodology

The rock samples used in this study were collected from a slope of the Wudongde Reservoir, which is situated at the junction of Luquan County, Yunnan Province and Huidong County, Sichuan Province, China. The samples are yellow sandstone with a yellowish-brown appearance. To minimize the influence of sample discreteness, all specimens were cored from a single rock block. Following ISRM recommendations, specimens were prepared with dimensions of Φ50 × 100 mm for uniaxial and triaxial compression tests, and Φ50 × 25 mm for Brazilian splitting tests. Each parallel test was repeated 3 times, and the median value was selected for analysis. After preparation, all rock samples were placed in a DHG series electric thermostatic blast-drying oven, dried at a constant temperature of 105 °C for 24 h, then taken out and cooled, and the WDC tests were performed.

The WDC treatment employed a natural immersion method. One cycle was defined as 24 h of immersion followed by 24 h of drying. The number of WDCs was set to 0, 5, 10, 15, and 20.

After 0–20 WDCs, characterization of the pore structure of yellow sandstone was carried out. Compared with conventional pore structure characterization methods such as mercury intrusion porosimetry and CT scanning, NMR technology has significant advantages including being non-destructive, non-invasive, rapid and efficient, without damaging the rock samples, and can directly provide pore size distribution. Thus, the interference of pore structure characterization on the inherent structure and mechanical properties of the rock was avoided. In this study, the tests were conducted using a MesoMR12-060H-60 NMR spectrometer manufactured in Suzhou, China (Suzhou Niumag Analytical Instrument Co., Ltd). The NMR testing was performed in accordance with the Chinese industry standard SY/T 6490-2014 [28]. Specification for measurement of rock NMR parameters in the laboratory. The test parameters are shown in Table 1.

After the NMR tests, rock mechanics tests were conducted on rock samples with different WDC times using an RMT-150B numerical control electro-hydraulic servo testing machine (Hohai University, Nanjing, China). The dimensions of the specimens for uniaxial compression tests and conventional triaxial compression tests are Φ50 × 100 mm, while those for Brazilian splitting tests are Φ50 × 25 mm. The confining pressure conditions for conventional triaxial compression tests were set to 5, 10, and 15 MPa. All three tests adopted displacement control loading with a loading rate of 0.001 mm/s.

**Figure 1:**
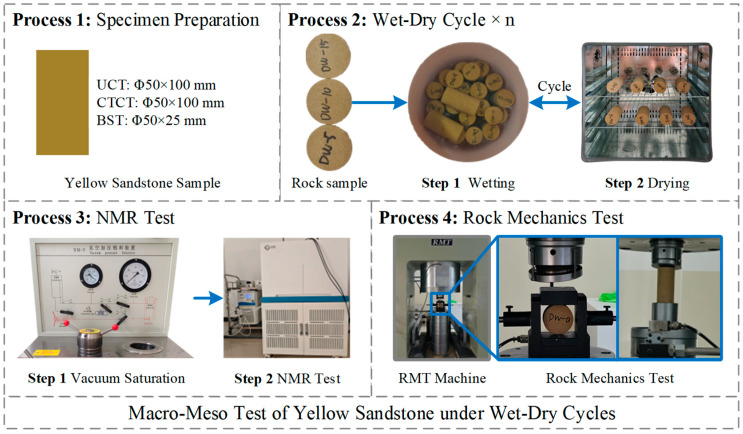
Macro–meso test process of yellow sandstone.

The overall process of the experiment is shown in Figure 1.

## 3. Test Results and Analysis

### 3.1. Distribution of NMR T2 Spectrum

Pore structure is a core determinant of the mechanical stability of yellow sandstone under seepage. Its pore size distribution directly determines the macroscopic mechanical properties of the rock. However, WDCs tend to induce pore evolution in sandstone and affect its functional stability. Therefore, it is necessary to reveal the variation law of the pore structure during this process through precise characterization techniques. First, NMR technology is used to obtain the pore size distribution characteristics of yellow sandstone under WDCs. One of the physical meanings of the NMR T2 spectrum is the pore size distribution, i.e., the proportion of the volume of pores at different scales to the total pore space. The transverse relaxation time T2 of NMR is affected by three relaxation mechanisms: free relaxation, surface relaxation, and diffusion relaxation, which can be expressed as [29]:

(1) $$\frac{1}{T_{2}}=\frac{1}{T_{2B}}+\frac{1}{T_{2S}}+\frac{1}{T_{2D}}$$

In the formula, $T_{2B}$ is the free relaxation time; $T_{2S}$ is the surface relaxation time; and $T_{2D}$ is the transverse relaxation mechanism under diffusion relaxation.

In rock mechanics tests, the transverse relaxation time T2 of fluids mainly depends on surface relaxation, and the other two mechanisms can be neglected [30]. Since surface relaxation is related to the specific surface area of rocks, the transverse relaxation time T2 can be expressed as:

(2) $$\frac{1}{T_{2}}\approx \frac{1}{T_{2S}}=\rho_{2}{\left(\frac{S}{V}\right)}_{\mathrm{pore}}=\rho_{2}\left(\frac{F_{S}}{r_{c}}\right)$$

In the formula, $\rho_{2}$ is the $T_{2}$ relaxation intensity on the surface of yellow sandstone particles; S is the pore surface area of yellow sandstone; V is the pore volume of yellow sandstone; (S/V)pore is the specific surface area of yellow sandstone pores; $F_{S}$ is the pore shape factor ($F_{S}=1$ for spherical pore model, $F_{S}=2$ for capillary bundle pore model, and $F_{S}=3$ for plate-like pore model); and $r_{c}$ is the pore radius.

The pore volume of yellow sandstone is calculated through Formula (2) and the porosity of yellow sandstone through Formula (3):

(3) $$n=\frac{V}{V_{s}}$$

In the formula, $V_{s}$ is the porosity of the yellow sandstone specimen and n is the volume of the yellow sandstone specimen.

**Figure 2:**
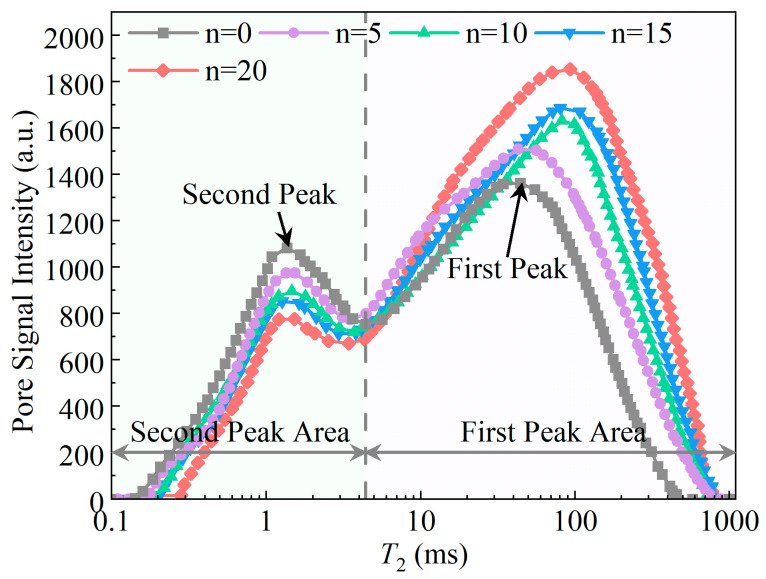
T2 curve of yellow sandstone specimens.

Figure 2 shows the T2 spectrum distribution curves of yellow sandstone with 0, 5, 10, 15, and 20 WDCs. Overall, the T2 spectrum distribution curves of yellow sandstone under the five wet–dry-cycle conditions exhibit a similar trend, with two distinct spectral peaks. It should be noted that according to Formula (1) the pore radius is proportional to the T2 relaxation intensity. Combined with the curve distribution characteristic that pore size increases with the increase in transverse relaxation time T2 [31], it indicates that there are two typical pore types in the yellow sandstone used in the test. Among them, the spectral peak near T2 ≈ 1 ms represents the small pore structure, while the peak near T2 ≈ 100 ms corresponds to the large pore structure.

**Figure 3:**
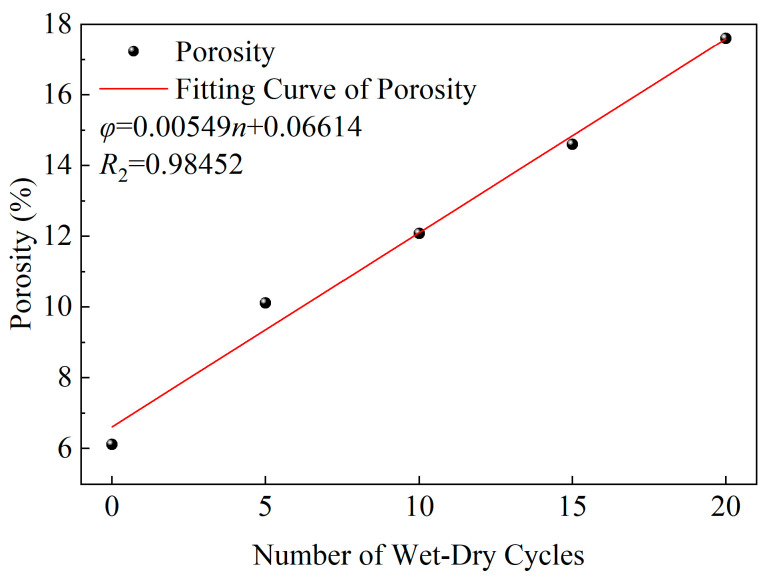
Relationship curve between porosity of yellow sandstone and the number of wet–dry cycles.

Existing studies have shown that the area of a rock’s T2 spectrum reflects changes in the size and quantity of internal pores, and is proportional to the size and quantity of the corresponding pores [32]. To quantitatively analyze the variation of the pore structure of yellow sandstone with the number of WDCs, the T2 spectrum area distribution and porosity of yellow sandstone after different WDCs were statistically analyzed, as shown in Table 2. It can be seen from Table 2 that both the total T2 spectrum area and the main peak area increase with the increase in WDCs, while the secondary peak area decreases correspondingly. Meanwhile, with the increase in WDCs, the peak positions of both small and large pore structures are slightly shifted towards longer T2 values. The above results indicate that the increase in the number of WDCs leads to a decrease in the proportion of small pores and an increase in the proportion of large pores inside the yellow sandstone. The porosity results show that the initial porosity of the yellow sandstone without WDC disturbance is 6.12%. As the number of wet–dry cycle increases, the porosity continuously increases, reaching 17.61% after 20 cycles, which is 187.75% higher than the initial porosity. The fitting result between porosity and the number of WDCs is shown in Figure 3. Figure 3 demonstrates that the porosity φ of the yellow sandstone exhibits a strong linear relationship with the number of WDC n.

### 3.2. Macroscopic Mechanical Test

Based on the shortcomings of existing studies, this study investigates the mechanical properties of yellow sandstone using Brazilian splitting tests, uniaxial compression tests, and triaxial compression tests.

**Figure 4:**
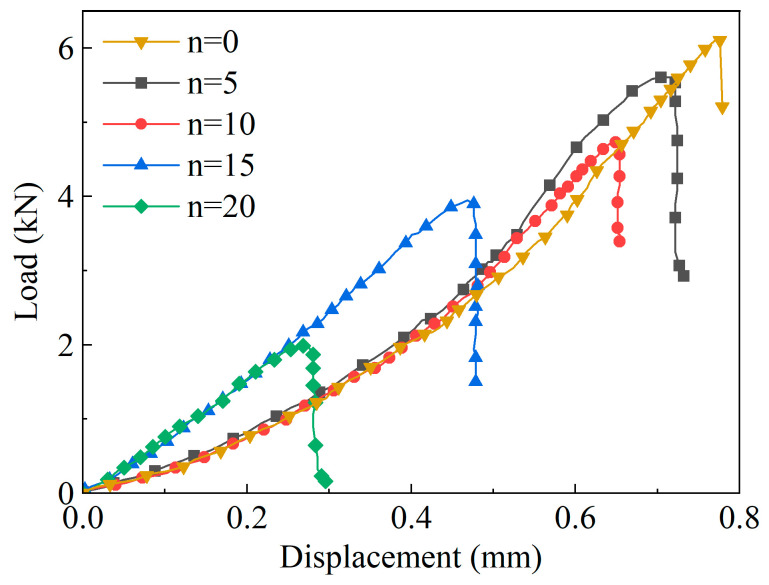
Brazilian splitting load–displacement curves under different wet–dry cycles.

The load–displacement curves obtained from the Brazilian splitting tests are shown in Figure 4.

As can be seen from Figure 4, the load–displacement curves of yellow sandstone under different numbers of WDCs all show a trend of first increasing and then decreasing. This is because, with the increase in load, the tensile strength of the rock gradually increases; after reaching the peak load, brittle failure occurs, leading to a cliff-like drop in the curve. When the number of cycles is 0, the peak load is 6.1 kN, and when it reaches 20 cycles, the peak load is 1.98 kN. It can be found that with the increase in WDCs, the peak strength of the rock shows a significant downward trend. This indicates that WDCs induce the development of internal microcracks and the deterioration of cementing materials in yellow sandstone, thereby greatly reducing its tensile strength.

**Figure 5:**
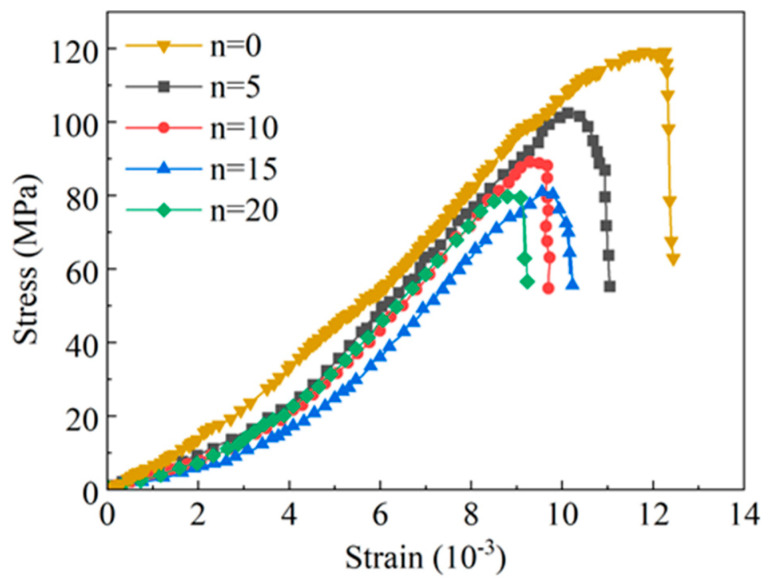
Uniaxial compression stress–strain curves under different wet–dry cycles.

Uniaxial compression tests were conducted on the prefabricated Φ50 × 100 mm yellow sandstone samples subjected to 0, 5, 10, 15, and 20 WDCs, respectively. The obtained stress–strain curves are shown in Figure 5.

As shown in Figure 5, the trends of stress–strain curves of yellow sandstone under different WDC numbers are nearly identical, indicating that the WDC process has no obvious influence on the mechanical response characteristics of the rock. All stress–strain curves undergo obvious pore-fracture compaction and closure stage, a long linear elastic stage, an unobvious yield stage and a plastic failure stage, suggesting that the 20-cycle WDC process has a slight effect on the elastoplasticity of yellow sandstone. By comparing the stress–strain curves of 0–20 WDCs, it can be seen that the variation amplitude of peak strength between groups with different WDC numbers gradually decreases with the increase in cycle number. This demonstrates that the damage capacity of the WDC process to the internal structure of yellow sandstone is limited.

**Figure 6:**
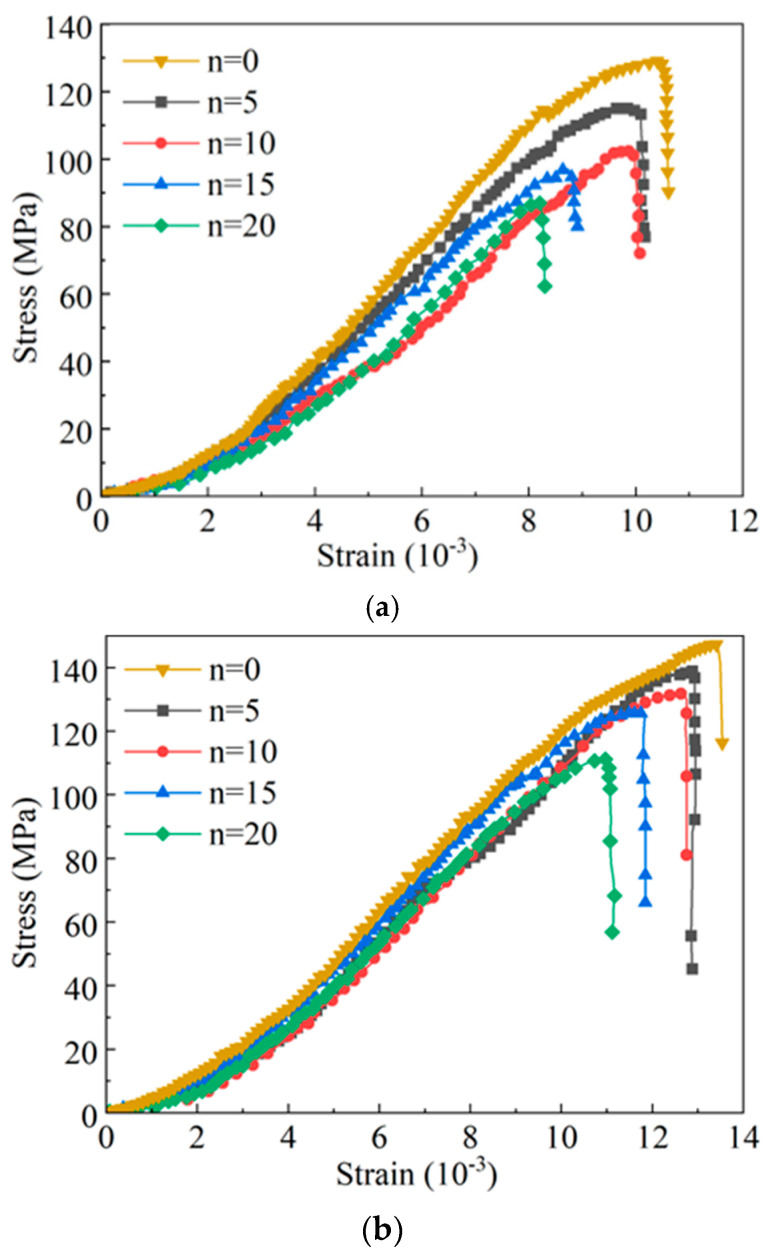

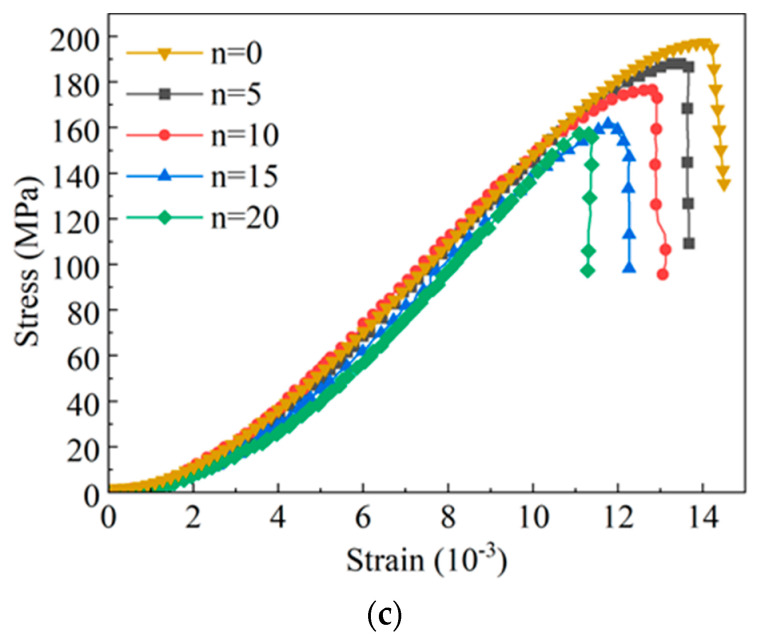
Conventional triaxial compression stress–strain curves under different wet–dry cycles and confining pressures. (**a**) Confining pressure 5 MPa. (**b**) Confining pressure 10 MPa. (**c**) Confining pressure 15 MPa.

Triaxial compression tests were conducted on yellow sandstone samples with different cycle times under various confining pressures (5, 10, and 15 MPa), and the resulting stress–strain curves are shown in Figure 6.

It can be seen from the conventional triaxial stress–strain curves under different confining pressures and WDC numbers that the stress–strain curves of yellow sandstone under different conditions are similar to those of uniaxial compression tests, all experiencing pore-fracture compaction and a closure stage, linear elastic stage, yield stage and plastic failure stage. Similar to the uniaxial compression results, WDCs have a weak modification effect on the brittle yellow sandstone under the same confining pressure. The peak strength gradually decreases with the increase in WDC number, indicating that WDCs cause varying degrees of deterioration to the internal load-bearing structure of the rock. Under the same WDC number, the peak strength increases with the increase in confining pressure, suggesting that confining pressure helps to enhance the overall WDC resistance of the rock. In addition, the plastic deformation stage of yellow sandstone gradually extends with the increase in confining pressure, indicating that confining pressure helps to restrict the lateral expansion and cracking of the rock, thereby improving its ductility.

## 4. Discussion

### 4.1. Influences of Wet–Dry Cycles on the Macroscopic Mechanical Parameters

**Figure 7:**
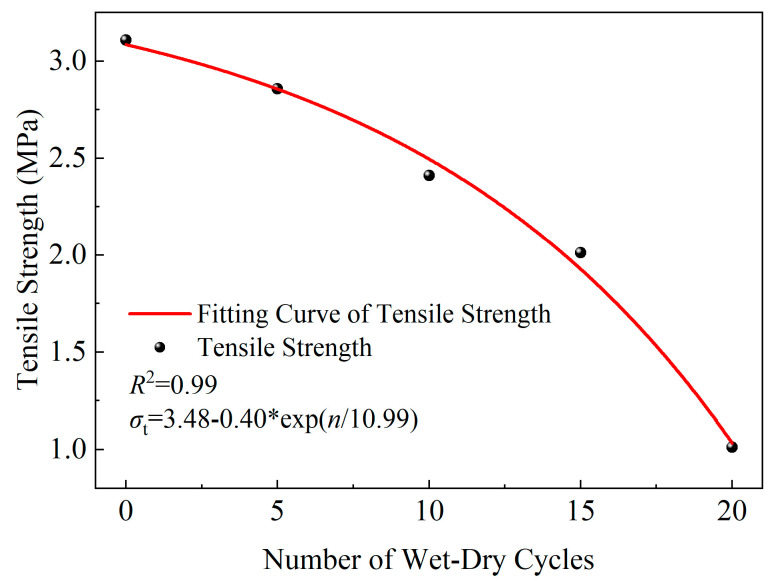
Relationship curve between wet–dry cycles and tensile strength.

Based on the Brazilian splitting test data of yellow sandstone obtained in Section 3, the influence of the WDC process on the tensile strength of the rock is analyzed. The tensile strength $\sigma_{t}$ is calculated using Equation (4), and the results are shown in Figure 7.

(4) $$\sigma_{t}=\frac{2P}{\pi Dh}$$

In the formula, P is the maximum load at specimen failure; D is the diameter of the disc specimen; and h is the thickness of the disc specimen.

As shown in Figure 7, the tensile strength of yellow sandstone exhibits a significant exponential decay with increasing number of WDCs. An exponential function model between the tensile strength of yellow sandstone and the number of WDCs was obtained through nonlinear fitting, with a correlation coefficient of 0.99, indicating that the model can quantitatively describe the strength deterioration process of yellow sandstone. During 0–20 WDCs, the tensile strength decreased from an initial 3.11 MPa to 1.01 MPa, with a strength deterioration rate of 67.52%. Meanwhile, the strength reduction from 0 to 10 cycles (0.7 MPa) is smaller than that from 10 to 20 cycles (1.4 MPa), suggesting that the damage of WDCs to the tensile strength of yellow sandstone tends to accelerate.

This tensile strength degradation stems mainly from two aspects: on the one hand, repeated water intrusion and evaporation cause uneven swelling and shrinkage of hydrophilic minerals (e.g., clay minerals) inside the rock, generating micro-shear stresses between mineral particles; on the other hand, water dissolves the cementing materials inside the rock, resulting in an increase in effective pore space.

It can be easily found from the NMR test data of 0–20 WDCs in Table 2 that the T2 spectrum area, which characterizes total porosity, increases from 244,745.99 to 704,304.15 with increasing WDC number, verifying the viewpoint that the effective pore space expands with WDCs.

**Figure 8:**
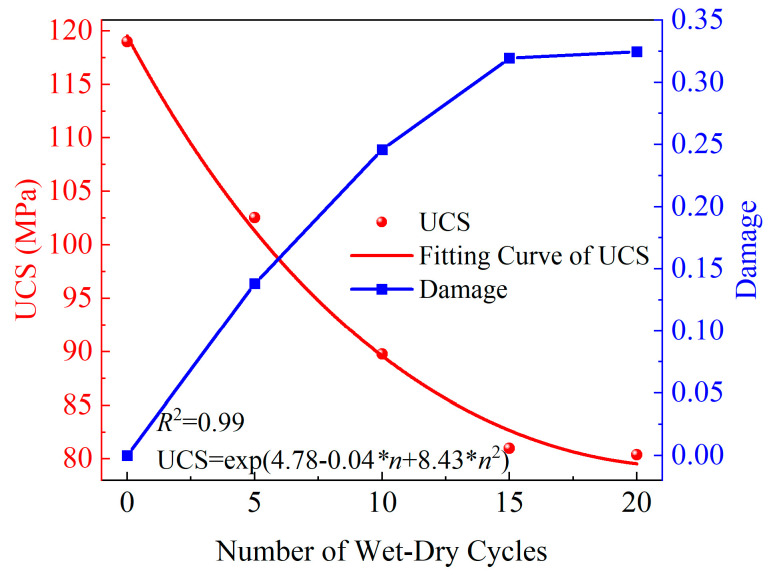
Relationship curve between rock damage factor and wet–dry cycles.

Based on the uniaxial compression test data of yellow sandstone in Section 3, the relationship between the strength damage factor of yellow sandstone and the number of WDCs was established with reference to the unconfined compressive strength of the specimen without WDCs, and the results are shown in Figure 8.

It can be seen from Figure 8 that the UCS of yellow sandstone shows an obvious nonlinear decay trend with increasing WDC number, while the corresponding strength damage factor exhibits a monotonically increasing characteristic. At equal intervals of WDCs from 0 to 20 cycles, the unconfined compressive strength of yellow sandstone decreases by 16.45 MPa, 12.79 MPa, 8.75 MPa, and 0.61 MPa, respectively, indicating that the effect of 0–20 WDCs on the compressive performance of yellow sandstone is limited. This conclusion is consistent with that reported by Ma et al. [33].

Based on the conventional triaxial compression test data of yellow sandstone in Section 3, the variation of peak strength with WDC number and confining pressure is summarized in Table 3.

**Figure 9:**
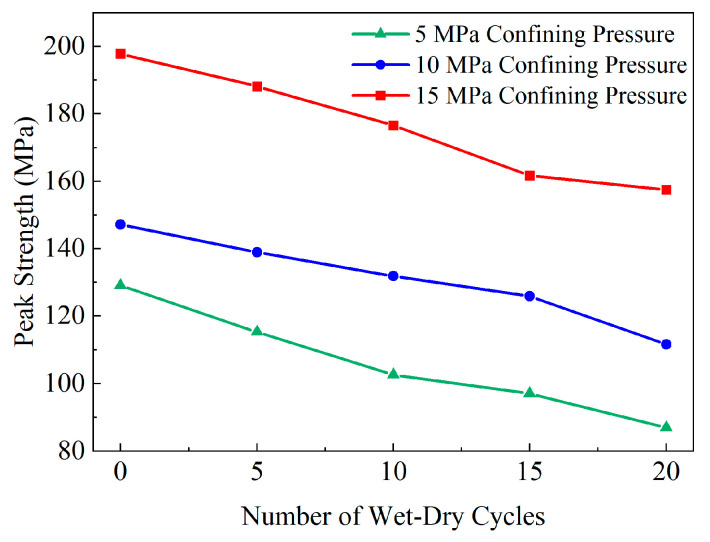
Relationship curve between peak strength and wet–dry cycles under different confining pressures.

**Figure 10:**
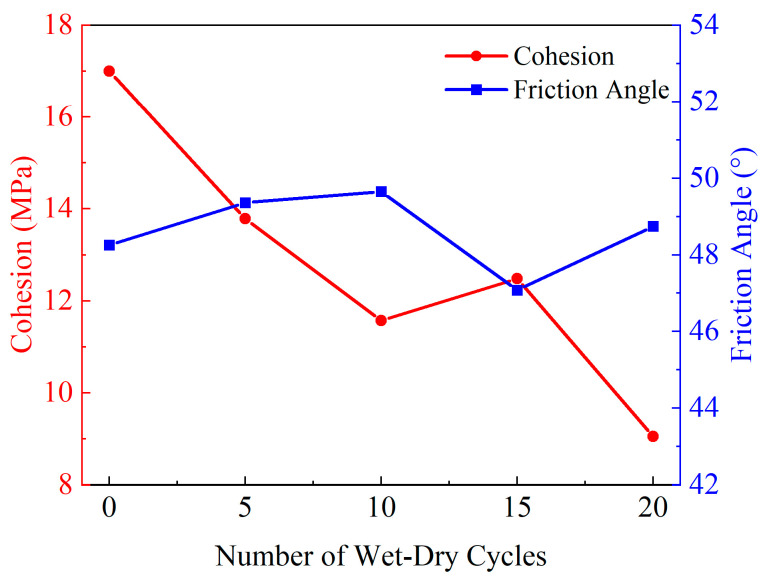
Relationship curve between cohesion, friction angle and wet–dry cycles.

Using the data in Table 3, the cohesion and internal friction angle of yellow sandstone under different WDC numbers were calculated by linear fitting based on the Mohr–Coulomb strength criterion (see Equation (5)), and the curves of cohesion and internal friction angle versus WDC number are plotted in Figure 9. Meanwhile, based on the data in Table 3, the curve of peak strength versus WDC number is plotted in Figure 10.

(5) $$\begin{array}{l} \sigma_{1}=a\times \sigma_{3}+b\\ a=\tan^{2}\left(45{}^{\circ}+\varphi /2\right)\\ b=2c\times \tan\left(45{}^{\circ}+\varphi /2\right) \end{array}$$

In the formula, $\sigma_{1}$ is the maximum axial stress at specimen failure; $\sigma_{3}$ is the confining pressure; φ is the internal friction angle of the specimen; and c is the cohesion of the specimen.

It can be seen from Figure 9 that the peak strength of yellow sandstone is dominated by the dual effects of confining pressure enhancement and WDC deterioration. For the confining pressure enhancement effect, the peak strength increases significantly with increasing confining pressure under any WDC number, indicating that confining pressure can effectively inhibit the initiation and propagation of internal cracks in the rock, increase the normal stress on internal fracture surfaces, and thus improve the shear bearing capacity of the rock. For the WDC deterioration effect, the peak strength shows an approximately linear decay trend with increasing cycle number.

Notably, the peak strengths from the Brazilian splitting test, uniaxial compression test, and conventional triaxial compression test show different evolution patterns, with decay rates characterized by acceleration, deceleration, and approximate linearity, respectively. These phenomena are closely related to the rock failure process. In the Brazilian splitting test, the rock is directly subjected to tensile stress and is most sensitive to internal micro-defects. In the uniaxial compression test, the microcracks inside the specimen undergo compaction and closure under compressive stress, which suppresses the generation of new cracks to a certain extent. In the conventional triaxial compression test, the rock is mainly constrained by confining pressure, which forces the closure of internal microcracks and restrains lateral expansion, thus weakening the deterioration effect of WDCs.

Figure 10 shows the variation of cohesion and internal friction angle with WDCs. At the microscopic level, the cohesion of rock is mainly composed of the chemical cementation force and van der Waals force between mineral particles, while the internal friction angle mainly depends on the mechanical friction properties between particles inside the rock.

With increasing WDC number, cohesion decreases sharply from 17 MPa (without WDCs) to 9.05 MPa at 20 WDCs, with a decrease in amplitude of 46.76%. This phenomenon indicates that the cementing materials inside yellow sandstone are dissolved and lost under repeated water soaking and erosion. In contrast to cohesion, the internal friction angle is relatively stable during the whole WDC process, with a maximum variation of only 2.57°, demonstrating that the pure physical process of WDCs has a slight effect on the overall internal structure of the rock.

### 4.2. Macro–Meso Damage Mechanism of Yellow Sandstone Under Dry–Wet Cycles

It can be found from the discussion in Section 4.1 that the increase in WDC number leads to varying degrees of reduction in tensile strength, uniaxial compressive strength and peak strength in conventional triaxial tests. Based on conventional triaxial compression test results, combined with the pore variation obtained by NMR tests, the correlation between the micropore-fracture structure and macro mechanical properties of yellow sandstone during WDCs is discussed.

Before the discussion, to eliminate the interference of initial pore differences among different specimens on the correlation analysis, the pore contribution degree is first defined, as shown in Equation (6).

(6) $$\begin{array}{l} \alpha =\frac{\Delta_{ni}}{f_{ni}}\\ \Delta_{ni}=\frac{S_{ni}-S_{n0}}{S_{n0}}\\ f_{ni}=\frac{S_{ni}}{S_{i}}\\ S_{i}=S_{1i}+S_{2i} \end{array}$$

In the formula, α is the pore contribution degree; $\Delta_{ni}$ is the relative change rate (RC) of pores; $f_{ni}$ is the pore proportion; $S_{ni}$ is the pore area at the i-th cycle, n=1 represents the pore area of micropores, and n=2 represents the pore area of macropores; and $S_{i}$ is the total pore area.

**Figure 11:**
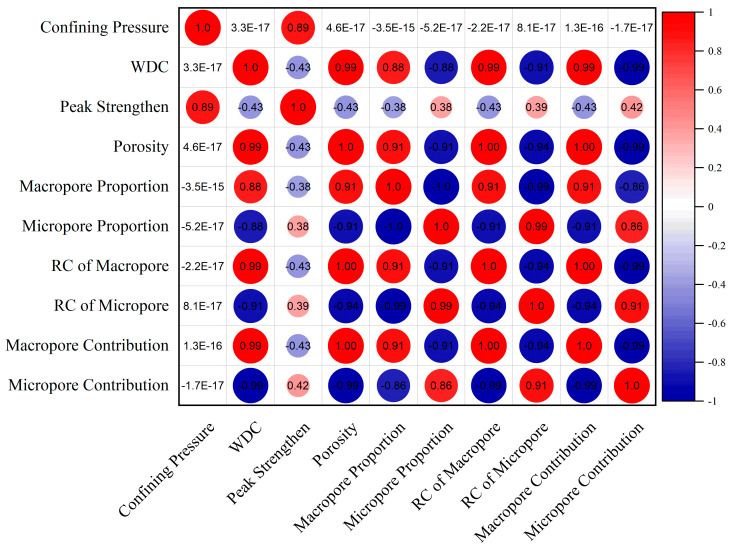
Pearson correlation heatmap of mechanical and pore structure parameters.

According to the calculation method of Equation (6), the correlations between different variables and peak strength are statistically analyzed, and the Pearson correlation heat map is shown in Figure 11.

It can be seen from Figure 11 that WDCs have an extremely strong positive correlation with macropore contribution degree (0.99) and an extremely strong negative correlation with micropore contribution degree (−0.99), indicating that with the aggravation of WDC damage, the pore structure evolution presents obvious heterogeneity. Macropores gradually become the dominant component of pore variation, and their contribution efficiency to the overall pore evolution continuously increases, while the contribution efficiency of micropores continuously decreases. The deterioration effect of WDCs on the pore structure of specimens is mainly dominated by the formation of macropore space.

The peak strength of yellow sandstone has a strong positive correlation with confining pressure (0.89), indicating that the higher the confining pressure, the higher the peak strength of the rock, which is consistent with previous studies. The peak strength has a moderate negative correlation with large pore contribution degree (−0.43), indicating that the development of large pore space induced by WDCs weakens the rock strength.

The variables affecting the peak strength are ranked as follows: confining pressure ≥ total porosity ≈ large pore contribution degree > small pore contribution degree, indicating that confining pressure plays a decisive role in rock stability during 0–20 WDCs, while the influence of pore structure variation is relatively weak.

**Figure 12:**
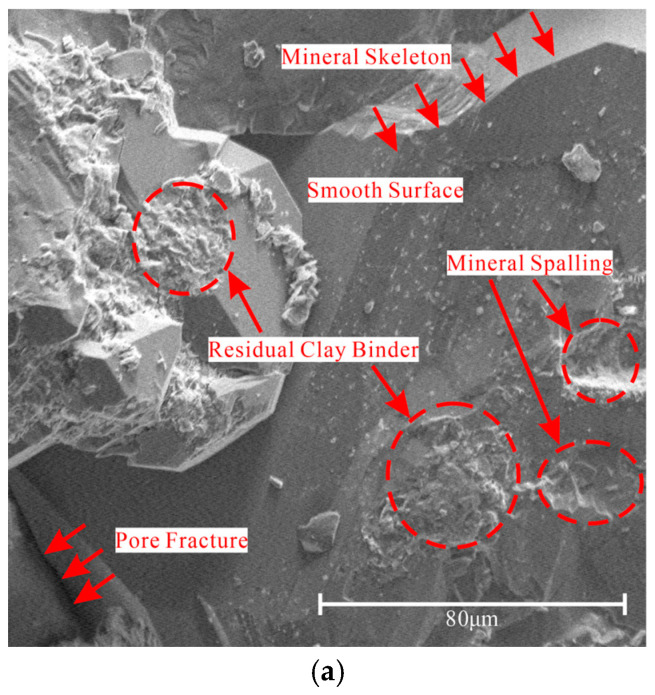

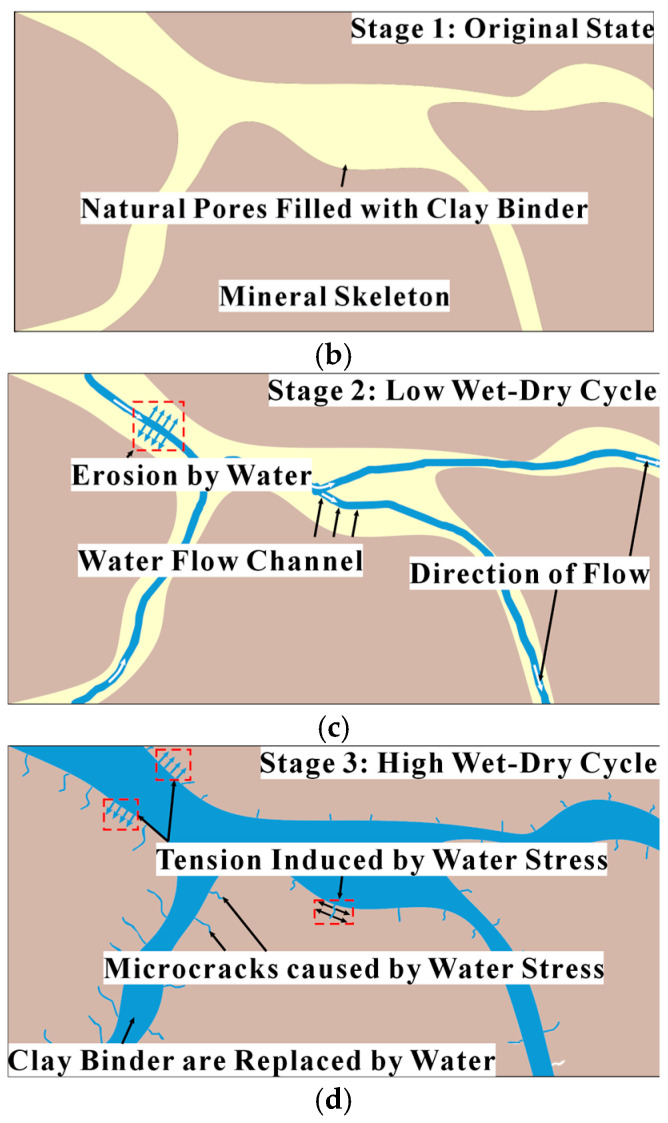
Schematic diagram of macro–meso damage mechanism of yellow sandstone under wet–dry cycles. (**a**) SEM image of yellow sandstone after wet–dry cycles. (**b**) Mesostructure in the original state. (**c**) Mesostructure under low wet–dry cycles. (**d**) Mesostructure under high wet–dry cycles.

Furthermore, the SEM results of yellow sandstone are shown in Figure 12a. It can be found from Figure 12a that most of the rigid mineral skeletons of yellow sandstone remain intact with a relatively smooth surface after WDCs. Residual clay cementing materials are sparsely distributed along the surface of mineral particles, and mineral spalling occurs in a small number of areas. The above phenomena indicate that WDCs mainly affect the clay cementing materials inside yellow sandstone but have little effect on the main skeleton structure. In addition, the shedding of clay cementing materials exposes part of the pores and fractures. The above effects jointly reflect the damage of WDC to the rock and reveal the essential mechanism of the mechanical property variation of yellow sandstone in the aforementioned studies.

Based on the test results obtained in this study and combined with the research conclusions of Ma et al. [34] and Wang et al. [35], the macro–mesoscopic damage mechanism of yellow sandstone under WDCs is summarized, as shown in Figure 12b–d.

As shown in Figure 12, Stage 1 represents the state unaffected by WDCs, Stage 2 represents the state with a low number of cycles and water saturation, and Stage 3 represents the state with a high number of cycles and water saturation. This study holds that the interior of yellow sandstone unaffected by WDCs is composed of a “skeleton structure + pore structure containing clay binder” (see Figure 12b). Overall, with the increase in WDCs, the clay binder in the pore structure is gradually dissolved and displaced by water.

In the stage with a low number of cycles and water saturation (see Figure 12c), the bearing capacity of the rock is provided by the “skeleton structure + clay binder accounting for most of the pore structure + water accounting for a small part of the pore structure”. At this time, the binder in the pores is carried away by water flow during WDCs, thereby reducing the contribution of binder to compressive strength and leading to a significant decrease in compressive strength.

In the stage with a high number of cycles and water saturation (see Figure 12d), the bearing capacity of the rock is provided by the “skeleton structure + pore structure filled with water”, and the rock skeleton contributes the vast majority of the bearing capacity at this time [36]. The rock skeleton structure is affected by water intrusion, which induces tensile stress near the original pore structure [37]. Meanwhile, the mineral cementation force in the skeleton is weakened due to dissolution [38], ultimately leading to an increase in rock porosity and microcrack damage in the skeleton structure. In addition, under the action of WDCs, the wet–dry difference inside the rock will cause stress concentration, which further aggravates rock damage.

## 5. Conclusions

This study integrated NMR technology and multi-scale mechanical testing to elucidate the deterioration mechanism of yellow sandstone under WDC. The following key conclusions are drawn:

(1) Macro–meso correlation: WDCs induce significant pore structure redistribution. The expansion of macropores directly correlates with the observed decay in rock strength. While tensile strength is highly sensitive to WDCs, confining pressure provides a “protective effect” by constraining lateral deformation and suppressing the growth of micro-defects.
(2) Damage essence: The degradation of yellow sandstone is fundamentally a process of “binder loss.” NMR and SEM analyses confirm that while the mineral skeleton maintains structural competence, the dissolution of clay binders and mineral spalling are the primary drivers of mechanical instability.
(3) The analysis mode and test results between WDC number, confining pressure, porosity parameters and strength are expected to provide strong support for the long-term stability evaluation of rock masses in engineering scenarios with wet–dry cycles, such as reservoir slope rock masses.
(4) Although up to 20 WDCs were carried out in this study, the long-term fatigue threshold of the rock mass remains to be determined. To further investigate this issue, it is necessary to increase the number of cycles and develop a multi-field coupling damage model combined with chemical damage, so as to better evaluate the long-term stability of the rock mass.
(5) In Section 4 it is concluded that the degradation of mechanical properties of the rock is caused by the dissolution of clay binder and partial damage to the mineral skeleton induced by WDCs. For future studies, it is recommended to conduct a comparative analysis of the damage effects of WDCs on rocks with different mineral compositions and degrees of weathering, so as to realize the rapid evaluation of mechanical properties of various rock types in engineering practice.

## Figures and Tables

**Table 1:** NMR test parameters.

Sequence Type	Sample Frequency	Dominant Frequency (MHz)	Radio Frequency Delay (ms)	Frequency Offset (Hz)	Digital Gain
CPMG	333.333	12	0.02000	195,096.53	3
90-degree pulse width (μs)	Analog gain (dB)	Entering radii data	Sampling number	Front-shift gear	Latency time (ms)
12.00	20	1	600,008	1	2500
Cumulative frequency	Pulse width of 180 degrees (μs)	Echo time (ms)	Number of echoes	-	
64	19.04	0.12000	15,000	-	

**Table 2:** T2 spectrum area and porosity after wet–dry cycles.

	T2 Spectral Area Data					Porosity
Number of Cycles	Total Area	First Peak Area	First Area Proportion	Secondary Peak Area	Secondary Peak Proportion	
0	244,745.99	241,074.02	98.50%	3671.97	1.50%	6.12%
5	404,941.78	402,355.93	99.36%	2585.85	0.64%	10.12%
10	483,088.09	480,525.20	99.47%	2562.89	0.53%	12.08%
15	583,951.94	581,779.60	99.63%	2172.34	0.37%	14.60%
20	704,304.15	702,282.86	99.71%	2021.29	0.29%	17.61%

**Table 3:** Peak strength of yellow sandstone under conventional triaxial compression.

Number of Cycles	Confining Pressure (MPa)	Peak Strength (MPa)
0	5	129.00
5		115.23
10		102.58
15		97.04
20		86.93
0	10	147.20
5		138.89
10		131.86
15		125.86
20		111.53
0	15	197.80
5		188.16
10		176.63
15		161.76
20		157.51

## Data Availability

The original contributions presented in this study are included in the article. Further inquiries can be directed to the corresponding author.
